# Development of Jellyfish (*Stomolophus* sp. 2) Gelatine–Chitosan Films: Structural, Physical, and Antioxidant Properties

**DOI:** 10.3390/gels11100836

**Published:** 2025-10-18

**Authors:** Dania Marisol Esparza-Espinoza, Francisco Rodríguez-Felix, Hisila del Carmen Santacruz-Ortega, Maribel Plascencia-Jatomea, Jesús Aarón Salazar-Leyva, Santiago P. Aubourg, Josafat Marina Ezquerra-Brauer

**Affiliations:** 1 Departamento de Investigación y Posgrado en Alimentos, University of Sonora, Hermosillo 83000, Sonora, Mexico; a213206430@unison.mx (D.M.E.-E.); francisco.rodriguezfelix@unison.mx (F.R.-F.); maribel.plascencia@unison.mx (M.P.-J.); 2 Departamento de Investigación en Polimeros y Materiales, University of Sonora (UNISON), Hermosillo 83000, Sonora, Mexico; hisila.santacruz@unison.mx; 3 Departamento de Ingeniería en Biotecnología, Universidad Politecnica de Sinaloa, Carretera Municipal Libre Mazatlán Higueras Km 3, Mazatlán 82199, Sinaloa, Mexico; jsalazar@upsin.edu.mx; 4 Department of Food Technology, Marine Research Institute (CSIC), C/Eduardo Cabello, 6, 36208 Vigo, Spain; saubourg@iim.csic.es

**Keywords:** jellyfish gelatine films, chitosan, antioxidant activity, physical properties, structural properties

## Abstract

The food packaging industry is inclined toward biodegradable films, and jellyfish hold significant potential for exploitation due to their extraordinary collagen content. Thus, the primary objective of this research was to develop an antioxidant gelatine-based film from the blue cannonball jellyfish (*Stomolophus* sp. 2) (JG), using chitosan (CH) and the casting method, with glycerol (GLY) as a plasticiser to improve film flexibility. The JG obtained through alkaline, heat, and dialysis treatment exhibited high in vitro antioxidant activity. A commercial chitosan acetic acid solution (1%) was added to a JG water solution (1%) and a commercial gelatine (CG) solution (1%) was employed as a control. The film’s mass ratio was 4:1:2 (JG:CH:GLY). The physical, chemical, thermal, mechanical, and antioxidant properties of the JG-CH and CG-CH films were compared; JG-CH showed higher solubility and thermal stability than CG-CH. The colour and light transmittance were similar; however, their tensile strength and elongation differed. Furthermore, JG-CH films exhibited a higher ABTS radical-scavenging capacity compared to CG-CH films. FTIR and ^1^H NMR spectra of the JG-CH films indicated excellent compatibility between the components, primarily due to hydrogen bonding. This study demonstrates that JG-CH films possess functional properties that make this material suitable for application as a biomaterial film for food.

## 1. Introduction

The widespread use of plastic packaging films derived from petroleum has created significant ecological challenges due to their non-biodegradable nature. To protect our environment, it is vital to limit and phase out plastic usage [1]. This has sparked a movement towards biodegradable packaging films and sustainable practices [2].

Biopolymers derived from renewable sources, including agricultural and marine origins, provide promising solutions in the form of compostable and edible packaging for food preservation that also exhibits bioactive properties, referring to characteristics that promote or influence biological activity. Gelatine, a protein derived from collagen, is one of the primary biomaterials used in the production of edible films [3]. Therefore, some studies have been conducted on the production of compostable materials based on marine gelatine, particularly those derived from jellyfish [4,5,6], to serve as an alternative to synthetic packaging materials. Although jellyfish-based gelatine is generally more expensive to produce than gelatine from land animals, it has several advantages, including a lower risk of zoonotic diseases, high biocompatibility, and a lower risk of inducing allergic reactions. The production of gelatine from land animals, although more economical, carries risks of disease transmission and may conflict with the dietary restrictions of certain religious groups. It also carries a risk of potential chemical contamination and a lengthy and costly production process [7]. While existing studies highlight a feasible solution to the ecological problems associated with non-biodegradable packaging films [5,8], the implementation of gelatine-like packaging has been limited so far due to its poor functional properties, which are attributable to the hydrophilic nature of gelatine [9].

Diverse tactics have been explored to improve the functional properties of jellyfish gelatine. Plasticising agents such as glycerol are used to improve flexibility and reduce fragility [10]. Moreover, because gelatine films are highly susceptible to moisture, mixtures with other biopolymers have been produced, mainly chitosan [11].

Chitosan is a ubiquitous polysaccharide derived from natural sources, primarily from the shells of crustaceans. Moreover, it is a cationic polymer that features free amino groups in a linear structure, characterised by a high density of positive charges. The presence of these amino groups has made chitosan one of the most versatile materials, finding applications in various areas, including film production [12]. Their film-forming attributes are correlated with their degree of acetylation or deacetylation, in conjunction with their molecular weight. A chitosan with a high deacetylation grade and high molecular weight offers the advantage of improving several physicochemical and bioactive properties of films. However, it also presents disadvantages, such as lower solubility due to its high molecular weight and potential processing difficulties resulting from its inherent rigidity [12]. After combining chitosan with gelatine, the resulting film exhibits improved properties, such as biodegradability, edibility, film-forming capacity, and biocompatibility [13]. In this context, the mixture of marine gelatine and chitosan has generated significant interest in food packaging applications, as they are both considered promising biodegradable materials for the development of edible films [13].

Chemical interactions between chitosan and gelatine occur through the presence of nucleophilic groups such as hydroxyl (OH) and amino (NH_2_) groups in chitosan and carboxyl (COOH) and NH_2_ groups in protein, as well as polarised heteroatom–hydrogen bonds. Among the most critical interactions are hydrogen bonds, ionic bonds, hydrophobic interactions, and van der Waals forces, with the former being the most representative [14]. These interactions lead to greater thermal stability in gelatine-based films [14,15]. Likewise, an increase in hydrophilic interactions may occur, leading to a reduced protein–protein association. Consequently, the viscosity of the mixture increases, promoting viscoelastic fluid-like behaviour, and elongation forces tend to decrease [16]. These modifications are crucial in packaging applications, as they enable superior handling and protection of food products [13].

However, research on the functional improvement of composite films based on gelatine from different marine sources is still considered insufficient. This encourages further studies to explore novel gelatines, such as jellyfish gelatine, in combination with chitosan, for the development of films with good functional properties.

The jellyfish has emerged as a highly profitable fishery product and an alternative for artisanal fishermen worldwide, mainly due to the decline of traditional fishing resources and the low cost associated with harvesting this marine organism [17]. This decline has significantly impacted the economic stability of artisanal fishermen, leading to numerous economic challenges [18]. However, jellyfish fishery presents a significant opportunity for positive change, with the potential to improve the financial conditions of these fishermen [19], especially with the use of jellyfish-derived biopolymers [10].

Sides of the American continents and zoan systematics consider *Stomolophus meleagris* [20] and *Stomolophus fritillaria* [21] as valid species within this jellyfish genus. However, the cannonball jellyfish living in the Gulf of California has changed from the meleagris species to as-yet-undefined species, called *Stomolophus* sp. 1 (located in the Gulf of Santa Clara, in the Upper Gulf of California) and *Stomolophus* sp. 2 (located throughout the southern Gulf of California) [22]. *Stomolophus* sp. 1 (brown colour) and 2 (blue colour) present a substantial commercial opportunity for those who rely heavily on fishing [19]. Between 2018 and 2024, the average catch of cannonball jellyfish (*Stomolophus* spp.) in Mexico was approximately 46,000 tons [23], and their gelatine extract exhibited good viscosity [24] and excellent antioxidant capacity [25]. Moreover, *Stomolophus meleagris* gelatine exhibited film-forming ability [26]. Therefore, the potential antioxidant film-forming capacity of blue cannonball jellyfish (*Stomolophus* sp. 2) captured in the Gulf of California, in combination with chitosan, should be investigated, with the goal of creating of a new antioxidant biofilm with potential applications in food packaging. Therefore, in this context, the aim of the present work was to investigate on a comparative basis the physical, light barrier, thermal, mechanical, and antioxidant properties of blue cannonball (*Stomolophus* sp. 2) gelatine–chitosan and commercial gelatine–chitosan composite films. Furthermore, to understand the interaction and the effect of individual compounds in the jellyfish gelatine–chitosan films, the compatibility of gelatine, chitosan, and glycerol was assessed through thermogravimetric analysis, Fourier-Transform Infrared Spectroscopy, and Proton Nuclear Magnetic Resonance studies. It was found that cannonball jellyfish gelatine films dislocated at a higher temperature and displayed higher antioxidant activity than commercial gelatine films. This research also demonstrated good compatibility between cannonball jellyfish gelatine and chitosan.

## 2. Results and Discussion

### 2.1. Properties of Jellyfish Gelatine (JG)

JG yield was 10.49 ± 0.18 g per 100 g of tissue, corresponding to a gelatine percentage of 45.6%. This yield aligns with the findings observed in the same species of jellyfish [27]. Still, it is higher than that reported for the jellyfish *Lobonema smithii* (26.4%) acquired under similar conditions [28]. The specific properties of the jellyfish species employed may have affected the degree of conversion of collagen to gelatine [29].

The gel and functional properties of any gelatine are closely tied to its amino acid composition [30]. This association highlights the remarkable characteristics that make gelatine a versatile ingredient across a multitude of applications. Notably, the sub-amino acids proline and hydroxyproline play a role in defining the functional properties of aquatic gelatine. The amino acid composition patterns of the studied JG were similar to those found in other studies involving jellyfish species captured in the Gulf of California [25,27]. The JG showed marked elevation in the levels of glycine (11.8%), arginine (4.5%), proline (4.5%), and hydroxyproline (3.0%). Additionally, the purity of the collagen could be confirmed because tryptophan and cysteine were not detected. The amino acid composition of the obtained gelatine, with diverse functionalities and levels of stability, could be achieved, particularly in terms of film formation, thereby enhancing its potential as a versatile ingredient in industrial applications.

The JG showed a higher ability to quench the free radical ABTS (IC_50_ of 250 μg mL^−1^) compared to commercial gelatine (IC_50_ of 1149 μg mL^−1^), chitosan (IC_50_ of 676 μg mL^−1^), and glycerol (IC_50_ of 1070 mg mL^−1^). Therefore, the results confirm our previous studies [25,27] showing that JG can prevent lipid oxidation and peroxyl radical formation through a chain-breaking reaction by scavenging free radicals [31] and donating a hydrogen atom [32]. Moreover, JG inhibits free radical-induced haemolysis (IC_50_ of 5.4 μg mL^−1^). An IC_50_ value higher than 10 μg mL^−1^ is considered to provide high protection for erythrocytes against radical damage [33]. These findings highlight the potential health benefits of JG and its applications in preventing oxidative damage in erythrocytes.

Once the antioxidant properties of the ingredients were identified, biofilms were produced, and the results shown below are based on a formulation established through preliminary tests. These tests involved the use of starting mixtures reported by several authors and adjusting them until a film was generated that was uniform, transparent, and could be removed from the mould.

### 2.2. Chemical Characterisation of Films

Gelatine and chitosan can form bonds in the presence of glycerol as a plasticiser [34]. Therefore, spectrophotometric analysis, FT-IR (Fourier-Transform Infrared Spectroscopy) and ^1^H-NMR (Proton Nuclear Magnetic Resonance) were performed to establish the interactions between jellyfish gelatine and chitosan.

#### 2.2.1. FT-IR

**Figure 1:**
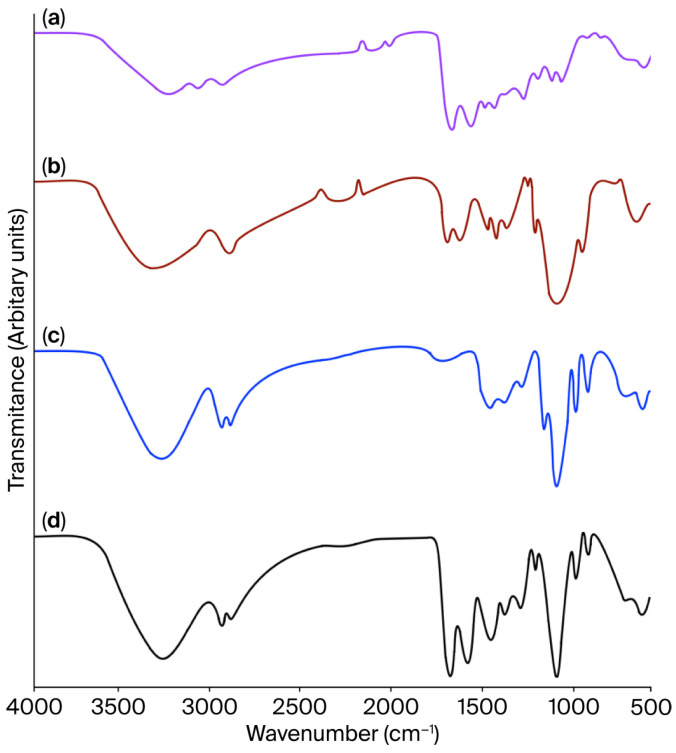
FT-IR spectra of (**a**) jellyfish gelatine, (**b**) chitosan, (**c**) glycerol, and (**d**) jellyfish gelatine–chitosan film. Film formulation: the gelatine (1%), chitosan (1%), and glycerol (1%), and the solution mass ratio was 4:1:2.

The FT-IR spectrum of JG-CH films (Figure 1) is comparable to those of JG, CH (Table 1), and glycerol (GLY) (Table 2). The IR transmittance spectrum of JG displays five major characteristic collagen peaks (Figure 1a) [35] and the wavenumbers of those peaks were as follows: 3247 cm^−1^ (Amide A) associated with N-H stretching; 2990 cm^−1^ (Amide B) linked to CH2 and NH3+ asymmetric stretching; 1635 cm^−1^ (Amide I) correlated to C=O stretching; 1585 cm^−1^ (Amide II) resulting from N-H and C-N torsional vibrations; and 1283 cm^−1^ (Amide III) corresponding to CH residual groups. Additionally, the N-H and C-OH wagging modes were observed between 682 and 562 cm^−1^ [35].

The CH spectra (Figure 1b) exhibit the peaks associated with Amide A (3260 cm^−1^), C-H stretching vibration of the methylene (-CH_2_) groups in the chitosan backbone (2835 cm^−1^), Amide I (1645 cm^−1^), and Amide II (1563 cm^−1^) [36]. Amide I and Amide II are associated with chitosan acetamide group residues [36]. Furthermore, at 1080 cm^−1^, skeletal motion characteristic of chitosan emerges, associated with pyranosidic and C-O-C groups. The -OH group in primary alcohol vibrations appears at 1480 cm^−1^. The -CH2 torsion and C-N tension are distinguished at 1406 and 1249 cm^−1^. Glycosidic stretching appears as the final peak between 675 and 596 cm^−1^ [36].

The GLY FT-IR spectra (Figure 1c) show peaks at approximately 3290, 2930, 1420, and 1110 cm^−1^, indicating -OH stretching, -CH stretching, COOH bending, and C-O stretching of primary and secondary alcohols [37]. Additionally, the skeletal vibration of the GLY structure is observed between 990 and 850 cm^−1^ [38].

The JG-CH film spectra displayed notable changes, including a slight shift in the Amide A peak from 3260 to 3175 cm^−1^, as well as a reduction in the -OH group peak and the Amide II peak in both JG and CH. These spectral alterations indicate interactions between the C=O group of JG and the N-H and O-H groups of CH. Additionally, the -OH groups were consumed during the interaction with GLY [39]. The observed behaviour was due to hydrogen bonding and physical interactions among gelatine, chitosan, and glycerol.

#### 2.2.2. Proton Nuclear Magnetic Resonance (^1^H-NMR)

**Figure 2:**
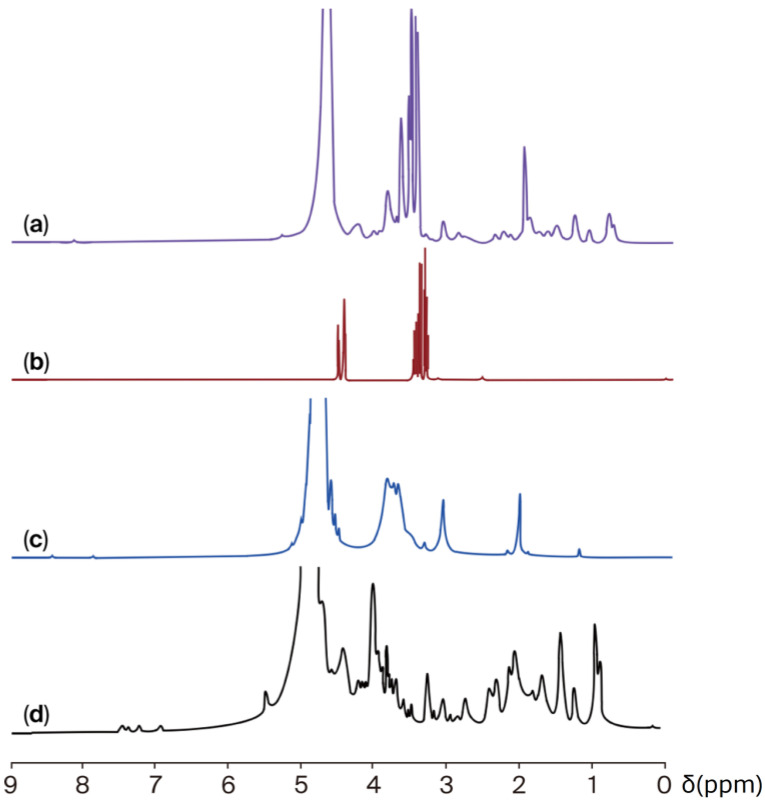
^1^H-NMR spectra of (**a**) jellyfish gelatine, (**b**) chitosan, (**c**) glycerol, and (**d**) jellyfish gelatine–chitosan film.

The chemical interaction between jellyfish gelatine and chitosan was also analysed using ^1^H NMR (Figure 2). The ^1^H-NMR spectrum of the jellyfish gelatine (Figure 2a) revealed peaks associated with the functional groups of various amino acid protons. The chemical shift around 0.5 to 1.5 ppm is likely attributed to the aliphatic carbon atoms of valine, leucine, and isoleucine. The signal of the methylene group (CH_2_) of glycine was observed at 4.2 ppm. The proton signals associated with proline were detected at 2.2 and 3.7 ppm. The signal found in 3.3 ppm is associated with hydroxyproline. The intense band at 4–6 ppm represents the “bound” water fraction, which interacts with the surface of collagen [40]. Chemical shifts at 8.6–8.1 ppm are indicative of the pyrrolidine ring [41].

The ^1^H NMR spectrum of chitosan (Figure 2b) indicated the presence of the three protons of N-acetylglucosamine (GlcNA), with a signal at 1.9 ppm. The H2 proton peaks of glucosamine residues (GlcN) were detected at about 3.1 and 3.2 ppm. In comparison, the peak at 3.1–3.2 ppm corresponds to the H2 protons of glucosamine (GlcN) residues. Additionally, the signals between 3.4 and 3.8 ppm represent the protons of D-glucosamine (H3–H6 protons) [42].

The ^1^H-NMR spectra of glycerol (Figure 2c) yield signals associated mainly with the different types of hydrogens in the molecule, including those of the CH_2_, CH, and hydroxyl (-OH) groups, at approximately 4.5, 3.4, and 3.3 ppm [43].

In the spectrum of JG-CH films (Figure 2d), a slight shift in chemical linking to protons present in leucine, proline, methionine, and hydroxyproline was detected. Additionally, no proton peaks (H2 and H3–H6) related to chitosan were identified. However, new signals were observed at approximately 3.4 and 4.5 ppm, denoting a change in the specific protons of chitosan (GlcNA and GlcN), attributed to a complex interaction between JG and CH involving electrostatic and hydrophobic interactions and hydrogen bond formation. These interactions are attributed to the presence of NH_2_, OH, and C=O groups in each ingredient used to produce the film [44]. Ultimately, the changes observed in the FT-IR and ^1^H-NMR signals indicate that the exposure of specific hydrophilic and hydrophobic amino acids in jellyfish gelatine, along with the amino groups of chitosan and the OH groups of glycerol, resulted in a fully miscible system. This comprehensive analysis not only informs us about the system’s miscibility but also about the primary interactions that occurred through hydrogen bonding between the NH_2_, C-H, and C-O groups [39].

### 2.3. Viscosity Values

**Figure 3:**
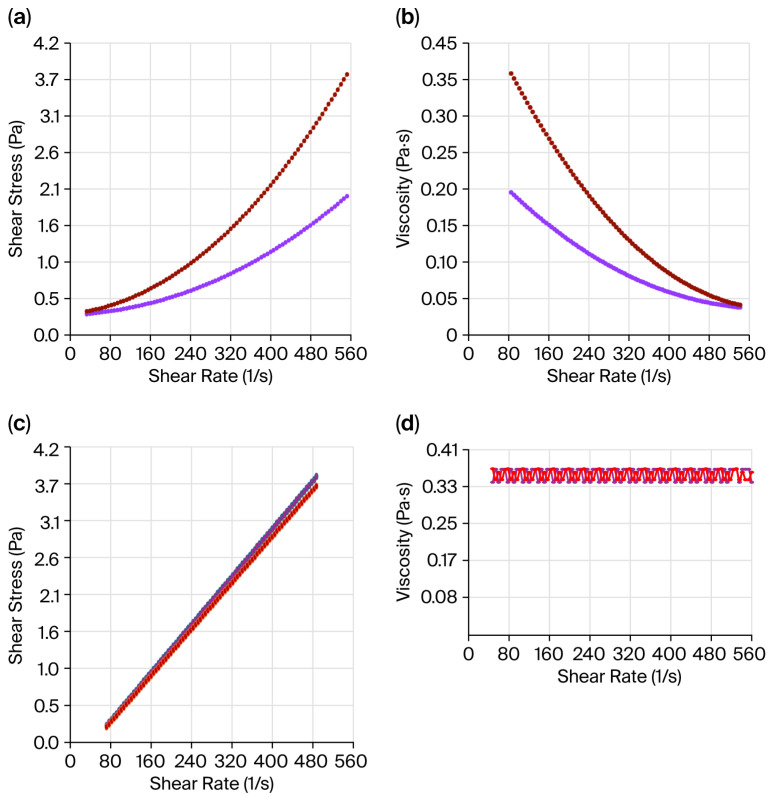
Jellyfish gelatine (—) and chitosan (—) solutions: viscosity flow curves versus shear stress (**a**) and apparent viscosity in response to shear rate (**b**). Jellyfish gelatine–chitosan film solution (—) and commercial gelatine–chitosan film solution (—): viscosity flow curves versus shear stress (**c**) and apparent viscosity in response to shear rate (**d**).

A crucial factor in determining the stability of biofilm particles and their potential applications is their viscosity. Typically, proteins and chitosan solutions behave as non-Newtonian fluids, as these materials do not exhibit a consistent interrelation between shear stress and shear rate [45]. The apparent viscosity of the JG and CH solutions increased as the shear rate increased, exhibiting non-Newtonian shear-thinning behaviour (Figure 3a). These results imply that the solutions exhibited a decrease in viscosity as the shear rate increased (Figure 3b) [46]. The above behaviour is in accordance with the previous report [47]. It is attributed to the orientation of dispersed molecules occurring as the shear rate increases, resulting in a decrease in internal friction due to a reduced successful interlinkage between molecules [48].

The biofilm-forming solution samples exhibited Newtonian behaviour (Figure 3c,d). This behaviour can be attributed to the crosslinking action involving gelatine and chitosan, whereby chemical crosslinking occurs between the functional groups of gelatine and chitosan (NH_2_ groups) and the OH groups of the plasticiser used, i.e., glycerol [47]. This process leads to the gradual replacement of the weak self-associated network of gelatine–chitosan with a stable covalent network, thereby enhancing the interaction of the biopolymers with water [48].

The jellyfish gelatine solutions were less viscous (0.274 ± 0.012 Pa·s) than the chitosan ones (0.738 ± 0.011 Pa·s), whereas no significant differences (*p* > 0.05) were observed between JC-CH (0.788 ± 0.012 Pa·s) and CG-CH (0.806 ± 0.008 Pa·s) films solutions. The viscosity observed in chitosan solutions can be attributed to the chitosan chains becoming fully extended and adopting a more linear conformation due to the strong repulsive forces between the positively charged amino groups, rather than those in gelatine [47]. Conversely, the higher viscosity values detected in the films can be linked to the increased number of interactions present in the analysed sample, reflecting the fact that the zero shear rate parameter is directly related to the number of bonds between the polymer molecules assessed [49]. These results align with those previously reported [47,49,50], and the viscosity measurements of the JG-CH solutions (0.788 Pa·s) reveal their potential for food coating applications [50].

### 2.4. Water Content and Stability of Films

Moisture content, which refers to the water molecules found between the free spaces within the biofilm network, and water stability are essential properties for the biofilm and its application. Understanding and controlling the moisture content is of utmost importance as it directly influences the physical properties of the biofilm [51]. Meanwhile, a material that is water-resistant to dissolution and ensures its durability is usually preferred. Even though the water content of both films was similar (*p* > 0.05) (JG-CH 18.0 ± 6.3% and CG-CH 14.0 ± 6.9%), JG-CH showed lower (*p* < 0.05) water stability (62.6%) than CG-CH (43%), which may be ascribed to differences in their amino acid composition, particularly jellyfish’s lower imino amino acid (proline and hydroxyproline) content (7.5 g 100 g^−1^) compared to land gelatine (31 g 100 g^−1^) [27].

### 2.5. Thickness, Appearance, and Optical Properties of Films

**Figure 4:**
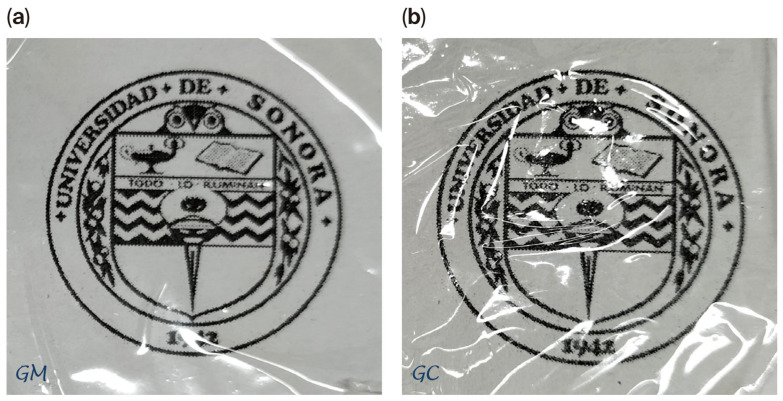
Photograph of the (**a**) jellyfish gelatine–chitosan and (**b**) commercial gelatine–chitosan films. Film formulation: the gelatine (1%), chitosan (1%), and glycerol (1%) mass ratio was 4:1:2.

The film thickness ranged from 0.025 to 0.029 mm for both films. The images (Figure 4) indicated that the aspect of both films was bright and translucent. Colour is a crucial indicator of overall appearance and consumer acceptability. The optical properties measured spectrophotometrically indicated that JG-CH films were opaquer than CG-CH films (Table 3). The increase in the opaqueness of jellyfish film could be attributed to the presence of pigment substances in jellyfish gelatine [52]. Furthermore, although the difference between the two films (ΔE*ab) was 1.61, suggesting that the films with jellyfish gelatine show a slightly more yellow and less reddish colour, the differences between these two films were not visible to the human eye, since their ΔE*ab value was less than three [53].

The quality of packaged food can be affected by ultraviolet–visible light (200–800 nm), mainly in the range of 200–300 nm, which induces oxidation and generates free radicals in food products [54]. Moreover, film transparency is a crucial feature of packaging materials, as high clarity is often desirable for food coatings or packaging applications. It was reported that when the light transmission rate of any film exceeds 90% at 600 nm, the film is transparent to the human eye [55]. The transmittance values (Table 4) of JG-CH and CG-CH were close to 90% at 600 nm, showing an opacity index (Abs_600_ thickness^−1^) of 1.89 (JG-CH) and 1.66 (CG-CH), implying that lower values mean higher transparency; however, no significant differences (*p* > 0.05) were detected among the films.

### 2.6. Stress–Strain Mechanical Properties (SSMPs) of Films

**Figure 5:**
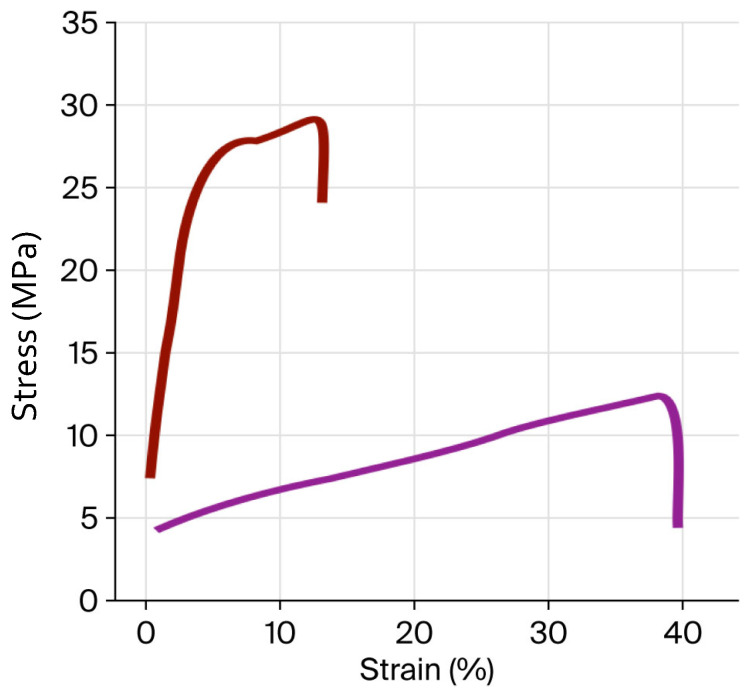
Stress–strain curves of Jellyfish gelatine–chitosan film (—) and commercial gelatine–chitosan film (—). Film formulation: the gelatine (1%), chitosan (1%), glycerol (1%) solution mass ratio was 4:1:2.

The SSMP values obtained from the stress–strain curves (Figure 5) of the films are summarised in Table 5. Although the stress curves of JG-CH and CG-CH films have a similar shape, the tensile stress (TS), elongation at break (εb), and elastic modulus (E) values were different. A lower TS value was detected in JG-CH films (12.07 MPa) compared with CG-CH films (29.23 MPa). This lower TS could be due to the lower generation of hydrogen bonds between functional groups of jellyfish gelatine and chitosan [56], attributed, as mentioned previously, to JG containing lower amounts of imino amino acids than gelatine obtained from land organisms [27,28]. In contrast, JG-CH displays higher εb (40.05%) and E (2.39 MPa) values than CG-CH, at 17.5% and 1.41 MPa, respectively. This implies that the interaction among JG-CH molecule chains releases tension in the film [56], resulting in enhanced flexibility. Moreover, based on the literature, the TS and εb values of JG-CH are comparable to those of elastic films [57], such as cellophane, low-density polyethene, and polypropylene, suggesting its potential application in food packaging.

Although the mechanical properties of the individual components were not evaluated in this study, other works have reported that the mechanical properties of films obtained from the gelatine-chitosan mixture present better properties when compared to films produced with the individual components [11,13,48,50]. Accordingly, it has been reported that pure chitosan films present higher TS but lower εb than gelatine-only films and composite [11,13,48,50]. To verify that under the conditions of this study, a similar behaviour is followed, further studies are required.

### 2.7. Thermogravimetric Analysis

**Figure 6:**
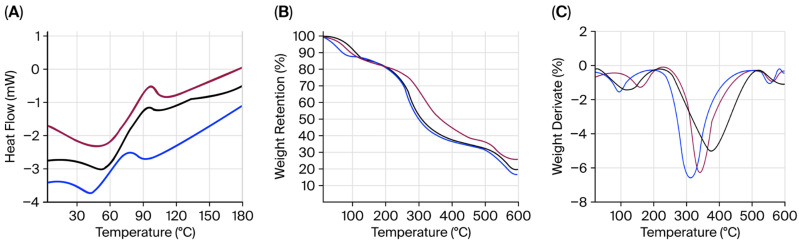
Thermal properties of jellyfish gelatine (—), commercial gelatine (—), and chitosan (—). (**A**) DSC; (**B**) TGA; (**C**) DTG. Film formulation: the gelatine (1%), chitosan (1%), glycerol (1%) solution mass ratio was 4:1:2.

**Figure 7:**
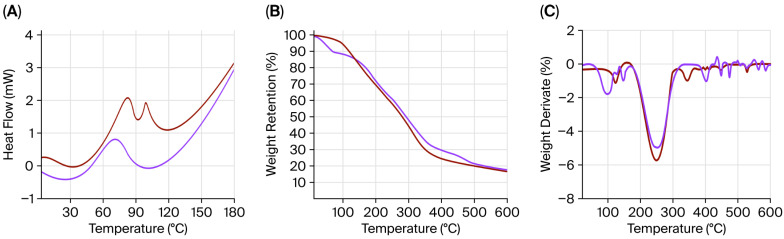
Thermal properties of jellyfish gelatine–chitosan film (—) and commercial gelatine–chitosan film (—). (**A**) DSC; (**B**) TGA; (**C**) DTG. Film formulation: the gelatine (1%), chitosan (1%), glycerol (1%) solution mass ratio was 4:1:2.

The thermal stability of the individual compounds (JG, CH, and CG) and the obtained films was studied using the scanning calorimetry technique (DSC), thermogravimetric analysis (TGA), and first derivative thermogravimetry (DTG). The DSC of the three individual compounds showed the glass transition temperature (Tg) as a step in the baseline, ranging from 54 to 73 °C (Figure 6A). The melting point (Tm) of JG is identified by one exothermic defined peak at about 88–98 °C, whereas that of CH was approximately 80–103 °C, and in the CG, it was detected at approximately 87–108 °C. The differences among gelatines are associated with variations in their amino acid profiles [58]. Similarly to individual compounds, both films (JG-CH and CG-CH) exhibited the Tg as a step in the baseline near 63.1 °C in the case of JG-CH and at around 93.2 °C for CG-CH (Figure 6A). Furthermore, the Tm of JG-CH was observed at 116.6 °C and at 157.2 °C for CG-CH; this indicates that the stable structure of the films was disrupted. The Tm value of the JG-CH films was approximately 13% lower than that of the CG-CH films (Figure 7A). The lower Tg and Tm of jellyfish gelatine compared to bovine gelatine are mainly associated with dissimilarities in their amino acid composition [57]. The lower imino amino acid content in jellyfish [27,28] facilitated easier molecular chain rearrangement during heating, inducing its lower Tg and Tm.

The TGA thermogram of individual components and films revealed three distinct degradation stages in the weight loss curve (Figure 6 and Figure 7). The first stage (103–160 °C) involved water evaporation and minor weight loss (4.3–11.9%), while the organic components decomposed in the second stage (329–369 °C), implying significant weight loss (≈75%) [59]. The disintegration of residual material that occurs beyond 599 °C corresponds to the third curve stage (Figure 6 and Figure 7) [60]. Furthermore, as shown in Figure 6B, JG presented a single degradation peak in the second stage. In contrast, the JG-CH film presented two independent peaks (Figure 7B), indicating the presence of two components [50]. DTG analysis of the film (Figure 7C) showed that at 399 °C, the JG-CH film retained 35.35% of its original weight, exceeding that of the CG-CH film (33%). Finally, above 600 °C, the JG-CH films retained approximately 20% more mass compared to the CG-CH films, indicating greater structural stability at higher temperatures in the JG-CH films, which contributed to improved thermal resistance properties.

### 2.8. Antioxidant Activity of Films

One process that affects ageing in cells is the entrapment of free radicals. Accordingly, this task estimated the capacity of the films to trap radicals (ABTS assay).

The two films exhibited scavenging activity against the ABTS radical. The IC_50_ values of the JG-CH films (406.5 μg mL^−1^) were lower (*p* < 0.05) than those of the CG-CH films (947.9 μg mL^−1^). The lower IC_50_ value of the JG-CH films indicated that this film has a higher capacity to trap the free radicals induced by ABTS.

The antioxidant activity in both films may be attributed to the unfolding of the collagen triple helix during the transformation of collagen into gelatine. Collagen disperses into aleatory peptide chains, and its electron-donating capability may be connected to its glycine, proline, and hydroxyproline content [61], as well as the positively charged amino groups of chitosan that would remain free, allowing a complementary effect stabilising free radicals and raising the film’s antioxidant capacity [59]. The higher antioxidant activity in the JG-CH film can be attributed to its unique amino acid composition, including a higher proportion of aromatic and hydrophobic amino acids [27]. Moreover, the percentage of inhibition of ABTS radicals in the JG-CH films (49.2%) was higher than that of a *Rhopilema esculentum* protein film with added wasabi extract (≈4 to 12%) [6].

## 3. Conclusions

In this study, gelatine extracted from the jellyfish *Stomolophus* sp. 2 was characterised and found to exhibit a high antioxidant capacity. A film was successfully obtained via a jellyfish gelatine–chitosan–glycerol casting method. The interactions between jellyfish gelatine, chitosan, and glycerol were primarily hydrogen bonding. Although the viscoelastic and optical properties and appearance of the biofilms based on jellyfish gelatine and commercial gelatine were similar, those obtained from jellyfish gelatine showed lower stability to moisture (approximately 60%) and tensile strength (approximately 40%) than those obtained from commercial gelatine. However, jellyfish gelatine-based biofilms were 59% more flexible, 20% more thermally stable, and 43% more capable of scavenging free radicals than those obtained from commercial gelatine. This innovative jellyfish gelatine–chitosan combination is intriguing, sparking future research. Although further evaluation is required and several challenges remain in commercialising the resulting material, the combination of jellyfish gelatine and chitosan enables the production of an innovative material for food preservation, and it could become an alternative to films made from synthetic materials. Nonetheless, it is necessary to define a specific combination of jellyfish gelatine and chitosan to establish the planned usages. Consequently, diverse blend ratios, in addition to further studies on antimicrobial properties and the applications as a food-protective film, among other uses, are essential and could form the basis of future research.

## 4. Materials and Methods

### 4.1. Materials and Reagents

Fifty specimens of blue cannonball jellyfish (*Stomolophus* sp. 2), a unique and rare species found in the Sea of Cortez, were selected for this study. This species, chosen for its unique gelatine properties and its potential for novel applications in food and materials science, presents an exciting opportunity for research and innovation. The jellyfish, measuring between 13 and 16 cm in length and weighing between 0.54 and 1.0 kg, were immediately placed on an ice bed after collection and transported to the laboratory. The mesoglea was then extracted from the specimens. Chitosan (from crab shells, 85% deacetylation and high molecular weight) and commercial gelatine (type B from bovine skin, 175 bloom) were obtained from Sigma Chemical Co. (St. Louis, MO, USA). Glycerol and all other reagents were of analytical grade and sourced from J.T. Baker (Mexico City, Mexico).

### 4.2. Gelatine Extraction and Analysis

The process of gelatine extraction, a meticulously performed and crucial step in this study, involved cutting the jellyfish mesoglea into small pieces and soaking them in 0.1 N NaOH at a ratio of 1:5 (*w*/*v*) for 24 h. This step was designed with utmost precision to break down the jellyfish tissues and extract the gelatine. The resulting protein solution was filtered through gauze and dialysed in water (4 °C) using a 10 kDa cellulose membrane and lyophilised. The collagen content in the sample was determined by measuring hydroxyproline (Hyp) and the overall protein content. Crude protein was quantified using a LECO FP-2000 Nitrogen Protein Analyser [62]. Hyp was analysed via high-performance liquid chromatography (RP-HPLC, Agilent Technologies, Santa Clara, CA, USA) [63].

The in vitro antioxidant activity of the JG sample was established by its protective effect against the radical ABTS^•+^ assay [64] and the radical AAPH on human erythrocytes [65]. The concentration of the sample required to inhibit 50% of ABTS or AAPH radicals (IC_50_) was determined using an inhibition curve constructed from absorbance values obtained from different concentrations of gelatine. Both assays were performed in triplicate.

The ability to quench the ABTS radical was also demonstrated in CG and CH under the same conditions mentioned previously.

### 4.3. Gelatine–Chitosan Films

The films were produced using the casting technique at 40 °C with mechanical stirring overnight [66]. The concentration of each ingredient and the proportion used to obtain the gelatine, chitosan, and glycerol-based biofilms were chosen based on studies conducted by other authors [6,10,11,34,50] and modified to ensure that the films were easily detachable from the mould used and exhibited a uniform and transparent appearance. Jellyfish gelatine (4% *wt*/*vol*), commercial gelatine (4% *wt*/*vol*), and chitosan (1% *wt*/*vol*) solutions were prepared separately. The lyophilised jellyfish gelatine and the commercial gelatine were dissolved in tridistilled water, and chitosan was dissolved in 0.1 M acetic acid. The plasticiser, glycerol (1%), was added to the film solution precursors under constant stirring at 350 rpm and 25 °C for 30 min to achieve a homogeneous mixture. The final gelatine–chitosan–glycerol mass ratio of the films was 4:1:2. The film-forming solution was then poured into Petri dishes (7 cm diameter) and kept at room temperature for 72 h, with humidity controlled using silica gel. The two obtained films were named JG-CH (jellyfish gelatine–chitosan) and CG-CH (commercial gelatine–chitosan).

### 4.4. Analysis

#### 4.4.1. Chemical Characterisation of Jellyfish Gelatine–Chitosan (JG-CH) Film

The interactions between jellyfish gelatine, chitosan, and glycerol were examined using FT-IR and ^1^H-NMR. Spectrophotometric studies were performed on the JG, CH, GLY, and JG-CH film in a dry state.

The FT-IR spectrum of dried samples (1 mg in 100 mg potassium bromide) was recorded at a temperature of 24 ± 1 °C using a Perkin Elmer spectrometer (Frontier MIR/FIR, Waltham, MA, USA). The spectra were collected over a range of 4000 to 400 cm^−1^ with a resolution of 4 cm^−1^, accumulating a total of 16 scans per spectrum. The system was purged with nitrogen gas during the acquisition of the spectra.

The ^1^H-NMR spectra were obtained at 24 ± 1 °C using a Bruker Avance 400 nuclear magnetic resonance spectrometer (Billerica, MA, USA), operating at 400 MHz. Lyophilised samples (1 mg) were dissolved in 0.5 mL of deuterated water (D_2_O) along with a 1% (*v*/*v*) deuterated hydrochloric acid solution (DCl 40% in D_2_O). Dimethylsilapentane sulfonic acid served as the reference, and the spectral window was set to 20 ppm.

#### 4.4.2. Viscosity

The viscosity was measured in the film-forming solutions [67]. The shear tests of JG, CH, JG-CH and CG-CH solutions were conducted in a stable state using an Anton Paar GmbH modular compact rheometer (MCR; model 102; Graz, Austria), which employed a concentric cylinder geometry. The shear rate range applied was 100 s^−1^ to 500 s^−1^ (25 °C). The average of 30 measurement points over 500 s was employed to express the viscosity results in Pascal seconds (Pa·s) [68].

#### 4.4.3. Water Content and Stability

The films’ water content and their stability in an aqueous medium were determined. First, the films (10 mg) were dried (100 °C, 24 h); then, they were placed in 50 mL of TRIS buffer (1 M, pH 7.2, containing 0.02% *w*/*v* sodium aside), and shaken for 24 h. Afterwards, the films were dried (100 °C, 24 h) and weighed to establish the content of insoluble dry matter [69]. Each sample was assayed in quintuplicate. The water content (Equation (1)) and degree of solubility (Equation (2)) were estimated [70].

(1) $$q=\left[\frac{Wf-Wi}{Wi}\right]\times 100$$

(2) $$W=\left[\frac{Wf-Wm}{Wf}\right]\times 100$$

where *Wi* is the initial weight of the sample (g), *Wf* is the weight of dry matter (g), and Wm is the weight of dry matter that did not dissolve after 24 h.

#### 4.4.4. Thickness, Appearance, and Optical Properties

Thickness measurements were carried out with a micrometre (Fowler 0–1″, Santa Clara, CA, USA) at nine random positions on each film. Only films with smooth surfaces and thickness with standard deviation less than 10% were selected, and some symmetrical sections were subjected to characterisation and averaged. These values were used in mechanical analysis.

The colour of the films was determined using a Hunter Lab ColorQuest II Spectrophotometer (Hunter Associates Laboratory, Inc., Reston, VA, USA). The colour parameters were expressed as lightness (L*), redness/greenness (a*), and yellowness/blueness (b*). The difference in colour (ΔE*ab) between films was calculated (Equation (3)) [71]. The values presented were the average of ten measurements.

(3) $${\Delta E}_{ab}^{*}=\sqrt{{\left(\Delta L*\right)}^{2}+{\left(\Delta a*\right)}^{2}+{\left(\Delta b*\right)}^{2}}$$

where ΔL*, Δa*, and Δb* are the differences between the colour parameters of the jellyfish gelatine–chitosan and commercial gelatine–chitosan films.

To measure the light transmittance and transparency of the films, they were cut into strips (4 cm × 1 cm) and placed on the inside wall of a plastic container (1 cm). The ultraviolet (UV) and visible light barriers of the films were measured between 200 and 800 nm [39]. Transparency was calculated using Equation (4).

(4) $$Transmitance=\frac{{Abs}_{600nm}}{film\;thickness}$$

#### 4.4.5. Stress–Strain Mechanical Properties

TS, εb, and E, were assessed by constructing a stress–strain curve via the ASTM D882 standard test [72]. Briefly, films were preconditioned at 25 °C and 50% RH in a desiccator for 40 h before analysis. Each film specimen (5 × 80 mm) was fixed between the separated grips (20 mm) of the texture analyser (United Model SSTM-5KN, Mexico City, Mexico) with the crosshead speed set to 10 mm s^−1^. TS (MPa) was calculated as the maximum load divided by the initial cross-sectional area of the sample and expressed in MPa. εb (%) was calculated as the ratio of the elongation at the point of the sample rupture and the initial length of the sample, multiplied by 100. E (MPa) was determined from the initial linear slope of the stress–strain curve. Results were expressed as means of at least five measurements.

#### 4.4.6. Thermal Properties

To determine the phase changes of JG-CH and CG-CH films and JG, CG, and CH compounds with temperature, the differential scanning calorimetry (DSC) technique was employed using conventional Perkin Elmer DSC equipment, model 8500. Approximately 7 mg of the sample was taken and sealed for heating at a rate of 10 °C/min from 25 to 200 °C under a nitrogen flow of 40 mL min^−1^.

Thermogravimetric degradation analysis was performed to investigate the temperature dependence of JG-CH and CG-CH films, as well as the starting compounds, including JG, CG, and CH. It was analysed using thermogravimetric analysis (TGA) and the derivative of weight loss (DTG) on a Perkin Elmer Pyris 1 instrument. Samples of approximately 5 mg were taken and subsequently heated to 600 °C at a heating rate of 10 °C/min under a nitrogen gas flow of 20 mL min^−1^.

#### 4.4.7. In Vitro Antioxidant Activity of Films

The in vitro antioxidant capacity of the films was evaluated using the ABTS radical-scavenging assay [64]. Briefly, 0.1 g of films was cut into small pieces, and the antioxidant compounds were extracted with a hydroalcoholic mixture under stirring overnight. After 30 min of rest, absorbance readings were taken at a wavelength of 734 nm. Each sample was assessed in triplicate.

The concentration of the JG-CH and CG-CH films (μg/mL) required to inhibit 50% of the ABTS radical (IC_50_) was determined by employing an inhibition curve established from absorbance values obtained at different concentrations of films and individual components.

### 4.5. Statistical Analysis

A completely randomised design was used as the experimental design, with treatment consisting of JG-CH films and CG-CH films. Statistics were determined using a one-way analysis of variance (ANOVA). Tukey’s comparison test established differences between means at a 95% significance level (α = 0.05). The statistical package used was SPSS (Version 20, 2011, SPSS Statistical Software, Inc., Chicago, IL, USA).

## Figures and Tables

**Table 1:** Assignment of FT-IR spectra of absorption bands of jellyfish gelatine (JG), chitosan (CH), and jellyfish gelatine–chitosan (JG-CH) film.

Assignments	JG	CH	JG-CH Film
N-H stretching, Amide A	3247	3260	3249
CH_2_ and NH_3_^+^ asymmetric stretching, Amide B	2950	–	–
C-H stretching vibration of methylene, -CH_2_ groups	2835	2835	2835
C=O stretching, Amide I	1635	1645	1630
N–H and C–N torsional vibration, Amide II	1585	1591	1545
CH residual groups, Amide III	1283	–	1285
Primary alcohol OH group	1480	1480	1480
-CH_2_ torsion and C-N tension vibration	–	1406–1249	–
Pyranosic and C-O-C groups	–	1080	–
N-H and C-OH out-of-plane bending	682–562	685–564	675–550

**Table 2:** Assignment of FTIR spectra of absorption bands of glycerol.

Assignments	Wavenumber (cm^−1^)
OH stretching	3290
CH stretching	2930–2870
Carboxyl group, C-OH bending	1420
Primary alcohol, C-OH stretch	1110
Skeletal vibration, C-C	990–850

**Table 3:** Colour properties ^1^ of jellyfish gelatine–chitosan (JG-CH) and commercial gelatine–chitosan (CG-CH) films.

Parameter	JG-CH	CG-CH
*L*	86.52 ^b^ ± 0.38	87.71 ^a^ ± 0.70
*A*	0.37 ^a^ ± 0.02	0.41 ^a^ ± 0.01
*B*	−2.15 ^a^ ± 0.05	−2.08 ^a^ ± 0.06
ΔE*ab ^2^	1.61 ± 0.32	

Film formulation: the gelatine (1%), chitosan (1%), and glycerol (1%) mass ratio was 4:1:2. ^1^ Mean value ± standard deviation from ten separate samples. ^2^ Differences in colour between the two films. Mean values followed by different letters (a, b) in each row indicate significant differences (*p* < 0.05).

**Table 4:** Transmittance values at different wavelengths and transparency ^1^ of jellyfish gelatine–chitosan (JG-CH) and commercial gelatine–chitosan (CG-CH) films.

Wavelengths (nm)	Transmittance ^2^ (%)	
	JG-CH	CG-CH
200	0	0
250	41 ± 1.3	42 ± 1.8
300	60 ± 4.1	68 ± 3.9
400	84 ± 3.3	90 ± 2.7
500	88 ± 2.8	92 ± 2.3
600	89 ± 3.8	92 ± 3.1
700	89 ± 2.4	92 ± 2.3
800	90 ± 1.8	92 ± 1.3
Opacity index (Abs_600_/thickness) ^3^	1.89 ± 0.16 ^a^	1.66 ± 0.09 ^a^

Film formulation: the gelatine (1%), chitosan (1%), glycerol (1%) solution mass ratio was 4:1:2. ^1^ Mean value ± standard deviation from five separate samples. ^2^ No significant differences were detected among films (*p* < 0.05). ^3^ The same letter means that no significant differences were detected among opacity index films (*p* < 0.05).

**Table 5:** Mean values of mechanical properties evaluated for jellyfish gelatine–chitosan (JG-CH) and commercial gelatine–chitosan (CG-CH) films.

Property	JG-CH	CG-CH
TS (MPa)	12.07 ^b^ ± 0.52	29.23 ^a^ ± 0.31
εb (%)	40.05 ^a^ ± 2.32	17.50 ^b^ ± 0.51
E (MPa)	2.39 ^a^ ± 0.14	1.41 ^a^ ± 0.19

Film formulation: the gelatine (1%), chitosan (1%), glycerol (1%) solution mass ratio was 4:1:2. Mean value ± standard error from five independent samples. Mean values followed by different letters (a, b) in each row indicate significant differences (*p* < 0.05).

## Data Availability

The data presented in this study are available in the article. Further information is available upon request from the corresponding author.

## Abbreviations

The following abbreviations are used in this manuscript:

JG	Jellyfish gelatine
CH	Chitosan
CG	Commercial gelatine
GLY	Glycerol
JG-CH	Jellyfish gelatine–chitosan–glycerol film
CG-CH	Commercial gelatine–chitosan–glycerol film
OH	Hydroxyl group
NH_2_	Amino group
COOH	Carboxyl group
ABTS	2,2′-azino-bis(3-ethylbenzothiazoline-6-sulfonic acid)
AAPH	2,2′-azobis(2-amidinopropane) dihydrochloride
IC_50_	Half-maximal inhibitory concentration
μ	Viscosity
W	Stability in aqueous solution
Δ*Eab*	Differences in colour
TS	Tensile stress
εb	Elongation at break
E	Elastic modulus
SSMP	Stress–strain mechanical properties
DSC	Differential scanning calorimetry
TGA	Thermogravimetric analysis
Tg	Glass transition temperature
Tm	Melting point
FT-IR	Fourier-transform infrared spectroscopy
^1^H-NMR	Proton nuclear magnetic resonance
C=O	Carbonyl group
C-H	Carbon hydrogen bond
C-O	Chitosan ether linkage and gelatine carboxylic group
NaOH	Sodium hydroxide
Hyp	Hydroxyproline
Mpa	Megapascal
